# Internet Addiction, Oral Health Practices, Clinical Outcomes, and Self-Perceived Oral Health in Young Saudi Adults

**DOI:** 10.1155/2020/7987356

**Published:** 2020-08-11

**Authors:** Asim Al-Ansari, Maha El Tantawi, Nasser AlMadan, Muhammad Nazir, Balgis Gaffar, Khalifa Al-Khalifa, Ali AlBaty

**Affiliations:** ^1^Department of Preventive Dental Sciences, College of Dentistry, Imam Abdulrahman Bin Faisal University, Dammam 31441, P.O. Box 1982, Saudi Arabia; ^2^Department of Pediatric Dentistry and Dental Public Health, Faculty of Dentistry, Alexandria University, Alexandria, Egypt; ^3^Ministry of Health, Dammam, Saudi Arabia

## Abstract

The study assessed the relationship between Internet addiction and oral health practices and clinical outcomes and whether this was affected by oral health perception. In 2017, a cross-sectional study included university students in the Eastern Province of Saudi Arabia. Questionnaires assessed demographic background, oral health practices (consuming sugar, tobacco use, and oral hygiene), perceived oral health, and Internet addiction. Caries experience and gingivitis were assessed using the World Health Organization criteria. The multivariate general linear analysis assessed the relationship between dependent variables (oral health practices, DMFT, and gingivitis) and exposure (Internet addiction). Data were available for 919 participants, 75.4% females, mean age = 19.8 years, and 1.6% with significant Internet use problem. The mean percentage of teeth with gingivitis was 8.5% and mean DMFT was 2.9. Among those with good perception of oral health and compared with participants with significant Internet use problem, average Internet users had lower consumption of sugar and tobacco (*B* = −6.52, *P* = 0.03 and *B* = −2.04, *P* = 0.03), better oral hygiene practices (*B* = 2.07, *P* = 0.33), higher DMFT (*B* = 2.53, *P* = 0.10), and lesser gingivitis (*B* = −15.45, *P* = 0.06). Internet addiction was associated with negative oral health practices and poor clinical outcomes among young Saudis. Holistic health promotion approaches need to address the negative impact of Internet addiction on health and oral health status for this at-risk age group.

## 1. Introduction

The use of the Internet has grown over the past few years especially among young people, and it is used for communication, socializing, searching for health information, gaming, and other purposes [1]. With this increase in its use, the term “Internet addiction” was used to describe the excessive nonessential use of the Internet that disrupts daily life, leading to personal distress or social, occupational, financial, or legal consequences [2]. Internet addiction also includes the inability to control the use of the Internet, continuous need for spending greater time on it, and sufferings from withdrawal symptoms [3]. Internet addiction is associated with mental and psychological symptoms [4] and negative health habits such as smoking, alcohol drinking, substance use [5], skipping meals [6], binge eating [7], not exercising, and not seeking medical care [6]. In addition, research also showed associations between Internet addiction and weight problems [7, 8], difficulty falling asleep, and increased waking up at night [6]. However, some debate exists about the nature of this excessive Internet use [9, 10], and the Diagnostic and Statistical Manual of Mental Disorders (DSM-5) does not include it as a formal disorder [11]. Young developed the Internet Addiction Test (IAT), which has been used in several studies of Saudi populations with the satisfactory performance [12–14]. A previous study showed Cronbach's alpha = 0.92 indicating excellent reliability of the IAT [15].

The association between Internet addiction and oral health has been assessed in several studies. Young adults who were Internet addicts reported tooth grinding during sleep [16]. Internet addiction was associated with a low frequency of tooth brushing, avoidance of dental checkups [8, 17, 18], and more self-reported gingival bleeding, tooth pain, and bad breath [18, 19]. Previous research suggested that excessive computer use was associated with less healthy periodontium, more bleeding on probing, and more untreated decay in permanent teeth among young adults from Poland [20]. However, the study addressed computer use in general and it is not known whether Internet addiction was associated with clinically assessed oral health outcomes [20].

Positive perception of health may be associated with better health behaviors leading to better health outcomes although this relationship has not been consistently reported [21]. Existing research focused on the association between Internet addiction and oral health practices. However, there is limited research about the impact of Internet addiction on the clinical outcomes of oral health.

In Saudi Arabia, 88.6% of the population had access to the Internet in 2019 [22]. Most Saudis are younger than 30 years of age, an age group with a high proportion of Internet use [23]. A closer look at the impact of Internet addiction on oral health in this population can help identify young adults at high risk of oral diseases and inform the development of health education programs. The aim of this study was to assess the relationship between Internet addiction and oral health practices and clinical outcomes among young Saudi adults. The study also aims to explore the differences in perception of oral health. We hypothesized that there was no association between Internet addiction and oral health practices and outcomes and whether participants had good or bad perception of their oral health.

## 2. Methods

The present cross-sectional study was conducted in Dammam, Saudi Arabia, from February to April 2017 after obtaining the approval of Imam Abdulrahman Bin Faisal University's (IAU) Institutional Review Board (IRB-2015-02-188). The study complied with the ethical guidelines of the Helsinki Declaration. A convenience sample of preparatory year (PY) students at IAU was selected. All students at IAU are admitted to PY program, which serves as a foundation year before students join their actual programs of study. Selected students must be 18 years or older and reported using the Internet. The sample size was estimated based on these assumptions: alpha error = 5%, beta error = 20%, percentage of individuals with unfavorable eating habits and Internet use problem = 21% and those with unfavorable eating habits, and no Internet use problem = 16% [24]. The minimum required sample size was 941. To make up for unusable questionnaires due to item nonresponse, all available students who met the inclusion criteria and consented were included because the permission given to approach preparatory year students was restricted to one semester only.

Dependent variables of the study were oral health practices: consumption of sugars, tobacco use, oral hygiene practices, and clinically assessed oral health: caries experience and gingivitis. The independent variable was Internet use. Several confounders such as age, gender, parental education, study track, and previous dental visits were assessed. Information about oral health practices and Internet use was collected using self-administered, validated questionnaires. The first questionnaire was the World Health Organization (WHO) oral health assessment questionnaire for adults [25]. We used the Arabic version of Khoshnevisan et al. [26] and culturally adapted it to ensure clarity of terms.

The questionnaire assessed confounders including personal background (age, gender, and parental education) and type of preparatory year studies track (health, engineering, science, law, education, and others). The questionnaire also assessed self-perceived oral health on a three-point Likert scale, poor, fair, and good, and regular dental checkup visits previous year. The next section assessed oral health practices: (a) frequency of tooth cleaning, (b) frequency of sugar consumption, and (c) frequency of tobacco use. The frequency of tooth cleaning was assessed on a six-point Likert scale ranging from never (0) to two or more times/day (5) for each of the four oral hygiene methods. A five-point Likert scale ranging from rarely (1) to several times/day (6) for six sugary products was used to evaluate the frequency of sugar consumption. The frequency of tobacco use was assessed using a seven-point Likert scale ranging from never (0) to several times/day (6) for five tobacco products. We also used the Internet Addiction Test (IAT) [27]. This questionnaire consisted of 20 statements with responses on a five-point Likert scale ranging from rarely (1) to always (5). Higher scores of IAT indicate greater Internet addiction problem. The original English version was translated into Arabic and then back-translated to ensure accuracy [28]. We piloted the questionnaire on 20 students whose responses were not included in the final analysis. In this pilot testing, face and content validity were assessed and the questionnaire was checked for clarity. The responses were also used to assess questionnaire reliability. The resulting Cronbach's alpha was 0.87, indicating excellent reliability.

Oral health was clinically assessed using the WHO methods and criteria for caries assessment including decayed teeth at the cavitation level (D), missing due to caries (M), and filled (F). Gingival inflammation was based on gingival bleeding after gentle probing defined as applying a force <20 g, which can be established by placing the tip of the probe under thumbnail and pressing till blanching [25].

PY students were approached by the study team during the breaks, and the study purpose was explained to them. Agreeing to join the study was considered as a verbal consent. Participants were asked to fill up paper-based questionnaires, which were collected in the same encounter. Full mouth clinical examination was conducted for all teeth using mobile equipment and disposable instruments under natural daylight [25]. Two calibrated examiners (Kappa ≥ 0.6) performed the clinical examination.

Three scores were developed: (1) oral hygiene practices score, (2) sugar score, and (3) tobacco score. The oral hygiene practices score was developed by multiplying the number of oral hygiene methods used by the participant by cleaning frequency. The score ranged from 0 to 20. The sugar and tobacco scores were the sum of the points of the frequency of all respective products and ranged from 1 to 36 and from 0 to 30, respectively. In all scores, higher values indicated greater frequency. The IAT score was categorized into average use (20–49), frequent use (50–79), and Internet use representing a significant problem (80–100) [27].

Caries experience (DMFT) score was the sum of the number of teeth that were decayed (D), missing due to caries (M), or filled (F) [25]. The percentage of teeth with gingival bleeding was also calculated. In bivariate analysis, we assessed the correlation between quantitative IAT score and age in years. IAT scores and the other study variables were assessed using *t*-test and one-way ANOVA test. A multivariate general linear model was used with stratification by perceived oral health to assess effect modification. The five dependent variables were (a) oral hygiene practices score, (b) sugar score, (c) tobacco score, (d) DMFT, and (e) percentage of teeth with gingivitis. The independent variable was the Internet addiction level. Internet addiction is also reported as problematic Internet use in the literature and researchers consider problematic Internet use a more appropriate term [29]. Hence, results show the use of the term “problematic Internet use.” We controlled confounders: age, gender, parental education, preparatory year track, and having regular checkups last year. The multivariate technique enabled us to assess the simultaneous association between all explanatory variables and the five dependent variables in one model. We calculated regression coefficients (B), 95% confidence intervals (CI), partial eta squared (*η*^2^) as measures of effect size, and *P* values. The significance level was set at 5%. IBM SPSS for Windows version 22.0 (IBM Corp., Armonk, N.Y., USA) was used for analysis.

## 3. Results

The questionnaires were distributed to 1900 students. Of those, 1830 answered the IAT and 1799 answered the WHO questionnaire. The response rate was 94.7%. Caries data were available for 1723 and gingivitis was assessed for 949. Therefore, further analysis was restricted to 919 students for whom complete data were available. On average, participants were 19.8 years old and most of them were females (75.4%). There were 23.9% and 21.6% from the health and engineering tracks, respectively. Of all participants, 49.3% had university-educated fathers and 40% had university-educated mothers. Only 10% visited the dentist regularly the previous year, and 15.1% had a poor perception of their oral health. Most participants were average Internet users (51.2%) and only 1.6% had significant Internet use problem (Table 1). Most participants (*n* = 556, 58.8%) had no gingival bleeding. The mean (SD) percentage of teeth with gingivitis was 8.5 (5.2). The mean (SD) DMFT = 2.9 (2.9), D = 0.9 (1.7), M = 0.1 (0.5), and F = 1.9 (2.5).

**Figure 1:**
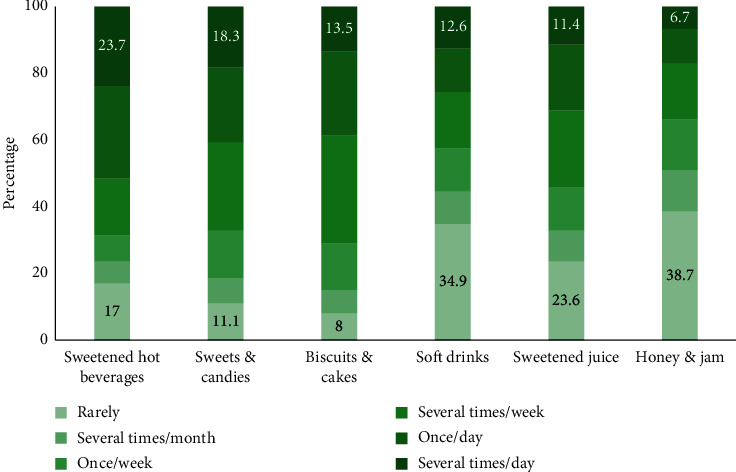
Frequency of consuming sweet products.

Most participants (*N* = 585, 62%) cleaned their teeth two or more times/day, 262 (27.8%) cleaned their teeth once daily, 49 (5.2%) 1-2 times/week, 21 (2.2%) 2-3 times/month, 21 (2.2%) rarely, and 5 (0.5%) never cleaned them. The majority (*N* = 906, 95.8%) used toothbrush and toothpaste and brushed twice/day using toothpaste 574 (60.9%). Only 224 (23.7%) used floss, 147 (15.6%) used miswak, and 143 (15.1%) used toothpicks. The mean (SD) oral hygiene practices score was 6.8 (3.7).  Figure 1 shows variation in the frequency of consuming sugary products among study participants. Sweetened hot beverages and honey and jam were used several times per day by 23.7% and 6.7% of participants, respectively. The mean (SD) sugar score was 21.0 (5.4).

**Figure 2:**
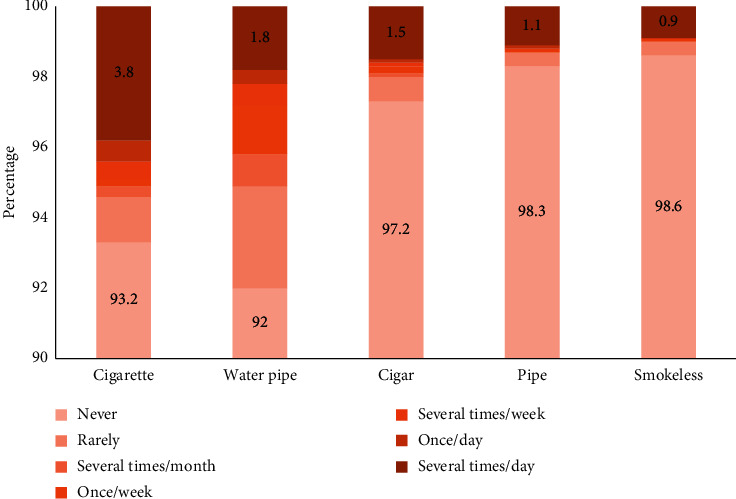
Frequency of using tobacco products.

Figure 2 shows the frequency of using tobacco products among study participants. The proportion of participants who never used tobacco ranged from 92% for water pipe to 98.6% for smokeless tobacco. The mean (SD) tobacco score = 0.8 (3.5).

Table 1 shows that, in bivariate analysis, males had significantly higher IAT score (mean = 51.99) than females (mean = 48.98) (*P* = 0.004). Similarly, participants with regular dental checkup during the last year had a significantly lower IAT score (mean = 46.69) than those without checkup (mean = 50.02) (*P* = 0.03).

Among participants with fair or good perception of oral health, average and frequent Internet use were associated with lower sugar scores than Internet use with significant problem (for fair perception, *B* = −5.90, *P*=0.003 and *B* = −5.54, *P*=0.006, for good perception, *B* = −6.52, *P*=0.03 and *B* = −5.26, *P*=0.09, Table 2). For those with poor perception, average and frequent Internet use were associated with nonsignificantly higher sugar scores (*B* = 4.62, *P*=0.40 and *B* = 6.44, *P*=0.24) than those with problematic Internet use. Average and frequent Internet use were associated with lower tobacco score compared to significantly problematic Internet use (*B* = −2.04, *P*=0.03 and *B* = −2.11, *P*=0.03). Oral hygiene practices score was nonsignificantly higher in average and frequent users compared to users with a significant problem among participants with fair or good perception (*P* > 0.05). In all oral health perception levels, DMFT was nonsignificantly higher among average and frequent users compared to users with significant problem (*P* > 0.05). The percentages of sites with gingivitis were nonsignificantly lower in average and frequent users compared to users with significant problems in all oral health perception levels (*P* ≥ 0.05). Partial eta squared (*η*^2^) values indicate small effect sizes in general ranging from <0.0001 to 0.02.

## 4. Discussion

The present study showed that, among participants with good and fair perception of oral health, average and frequent Internet users had less negative oral health practices (sugar and tobacco consumption) and more positive oral health practices (oral hygiene) than participants with problematic Internet use. However, in participants with poor oral health perception, average/frequent Internet use was associated with more sugar consumption. Across different oral health perceptions, caries experience was higher in frequent/average Internet users while gingivitis was lower in those with significant Internet use problem. Our findings partially support the study hypothesis among those with good/fair perception of oral health. This has implications for risk assessment of caries and gingivitis and the development of health education programs for Internet users.

In the present study, 1.6% of participants had problematic Internet use. This level is lower than that reported among Taiwanese (15.3%) [30], Chinese (11%) [31], and Turkish (9.7%) [32] college students. Similar prevalence estimates were reported among female college students in AlJouf, Saudi Arabia (1.9%) [13], but higher among Saudi students in Taif University (4%) [14]. The differences between these studies and ours may be attributed to methodological variations.

In the present study, among participants with good/fair perception of oral health, those with problematic Internet use had a greater consumption of sugar than those with average/frequent Internet use. Our findings agree with the results of studies of adolescents conducted in Korea [6], multiple European countries [33], Egypt [34], and Canada [35] reporting higher likelihood of consuming sugary snacks and beverages. Our study adds to the existing body of knowledge by documenting this association in young adults as well.

In addition, our study found that participants with a good perception of oral health who were average or frequent Internet users had lower tobacco scores than those with problematic Internet use. This agrees with studies from Korea [5, 6] and Taiwan [36] which reported greater percentages of tobacco users, lifetime smokers, or future smokers with problematic Internet use compared with moderate Internet users. It also agrees with a study of Iranian university students reporting a higher problematic Internet score among smokers than nonsmokers [36] but disagrees with another study showing no association between smoking and Internet addiction among young Vietnamese [37]. The difference may be explained by the higher prevalence of Internet addiction (21.2%) among Vietnamese participants [37].

Our study showed more frequent oral hygiene practices among participants with fair/good perception of oral health who were average/frequent Internet users than those with problematic Internet use. This is in agreement with two studies of Korean adolescents which reported that problematic Internet use was associated with less frequent tooth brushing [17, 18]. This negative effect on oral health practices and clinical outcomes may be attributed to disturbed sleeping patterns, stress, fatigue, and reduced motivation for self-care [38]. On the other hand, our finding disagrees with a study of Thai university students reporting no relationship between brushing twice daily and the time spent on the Internet [8]. The difference in these studies may be attributed to how researchers defined the Internet use problem which may not necessarily involve an addiction problem.

In the present study, participants with a poor oral health perception and Internet addiction had a lower frequency of sugar consumption than those with average/frequent Internet use. This may be attributed to compensatory health beliefs where individuals compensate for one type of unhealthy behavior by engaging in other healthy behavior/s [39]. Thus, participants tried to make up for Internet addiction by reducing the harmful sugar consumption and thus adopting less harmful dietary habits whereas moderate Internet users with poor oral health perception may have consumed sugar more frequently.

The present study showed that the gingival condition was better among those with average/frequent Internet use whereas their caries experience was higher than those with problematic Internet use. The greatest portion of DMFT (2.9) consisted of filled teeth (1.9, i.e., 65.5%). Therefore, the observed association reflects greater exposure to dental care rather than higher level of untreated decay among participants with average/frequent Internet use. The significant association with some oral health practices compared to the nonsignificant relationship with clinical outcomes suggests that more time is needed for Internet addiction to reflect on clinical outcomes.

The present study is limited by its cross-sectional design which cannot support causality and can only suggest associations which can be assessed in longitudinal cohort studies. The IAT does not assess the purpose of Internet use. Hence, this gives equal importance to playing games and using educational resources which may have different impacts on oral health. The strength of the study lies in the clinical assessment of oral health outcomes which fills a knowledge gap regarding the oral health effects of Internet addiction. The study also includes male and female students in a number big enough to allow adjustment for confounders. Future longitudinal studies are needed to assess the long-term impact of Internet addiction on oral health and whether this impact persists into adulthood. In addition, future studies are needed to assess the effect of oral health education provided over the Internet on reducing the adverse impact of Internet addiction on oral health outcomes.

## 5. Conclusion

The present study showed that Internet addiction was adversely and significantly associated with oral health practices and poorer clinical outcomes. Young Saudi adults with problematic Internet use may be at higher risk of oral diseases. Comprehensive health education interventions should promote healthy practices and moderate Internet use in this high-risk group.

## Figures and Tables

**Table 1:** Personal, socioeconomic, and dental characteristics of participants.

Factor		Statistic	IAT score	
			Mean (SD)	*P* value
Gender	Male: *n* (%)	232 (24.6)	51.99 (14.69)	0.004^*∗*^
	Female: *n* (%)	712 (75.4)	48.98 (13.69)	

Track	Health: *n* (%)	224 (23.9)	48.70 (14.94)	0.18
	Engineering: *n* (%)	87 (9.3)	52.30 (12.79)	
	Science: *n* (%)	203 (21.6)	48.93 (12.93)	
	Other: *n* (%)	424 (45.2)	49.88 (14.03)	

University-educated father	Yes: *n* (%)	464 (49.3)	49.38 (13.45)	0.44
	No: *n* (%)	478 (50.7)	50.08 (14.52)	

University-educated mother	Yes: *n* (%)	376 (40)	49.85 (13.82)	0.79
	No: *n* (%)	565 (60)	49.60 (14.12)	

Regular checkup last year	Yes: *n* (%)	94 (10)	46.69 (12.97)	0.03^*∗*^
	No: *n* (%)	846 (90)	50.02 (14.07)	

Perceived oral health	Poor: *n* (%)	139 (15.1)	51.29 (13.58)	0.16
	Fair: *n* (%)	405 (43.9)	49.86 (13.52)	
	Good: *n* (%)	379 (41.1)	48.74 (14.36)	

Problematic Internet use	Average: *n* (%)	484 (51.2)	—	
	Frequent: *n* (%)	447 (47.3)		
	Significant problem: *n* (%)	232 (24.6)		

^*∗*^Statistically significant at *P* < 0.05. *P* value: Pearson correlation for age, ANOVA for track and perceived oral health, and *t*-test for the remaining factors. The mean (SD) age of the sample was 19.8 (1.4) years and Pearson correlation coefficient between age and IAT score was 0.02 (*P*=0.60).

**Table 2:** Association of Internet use with oral health practices and clinical outcomes stratified by the perception of oral health.

Perceived oral health	Problematic Internet use		Sugar score	Tobacco score	Oral hygiene practices score	DMFT	Percentage of teeth with gingival bleeding
Poor	Average vs. significant problem	Regression coefficient (95% CI)	4.62 (−6.15, 15.40)	−1.75 (−13.69, 10.18)	0.28 (−7.78, 8.33)	2.41 (−4.08, 8.90)	−6.51 (−47.82, 34.80)
		*P*	0.40	0.77	0.95	0.46	0.76
		*η* ^2^	0.006	0.001	<0.0001	0.005	0.001
	Frequent vs. significant problem	Regression coefficient (95% CI)	6.44 (−4.42, 17.29)	−2.46 (−14.48, 9.56)	−0.32 (−8.43, 7.80)	2.42 (−4.12, 8.96)	−7.01 (−48.62, 34.60)
		*P*	0.24	0.69	0.94	0.47	0.74
		*η* ^2^	0.01	0.001	<0.0001	0.005	0.001

Fair	Average vs. significant problem	Regression coefficient (95% CI)	−5.90 (−9.83, −1.98)	−0.86 (−3.25, 1.53)	0.86 (−1.77, 3.49)	0.62 (−1.48, 2.72)	−4.25 (−13.86, 5.35)
		*P*	0.003^*∗*^	0.48	0.52	0.56	0.39
		*η* ^2^	0.02	0.001	0.001	0.001	0.002
	Frequent vs. significant problem	Regression coefficient (95% CI)	−5.54 (−9.44, −1.64)	−0.79 (−3.16, 1.58)	0.48 (−2.13, 3.09)	0.62 (−1.47, 2.71)	−6.14 (−15.68, 3.40)
		*P*	0.006^*∗*^	0.51	0.72	0.56	0.21
		*η* ^2^	0.02	0.001	<0.0001	0.001	0.004

Good	Average vs. significant problem	Regression coefficient (95% CI)	−6.52 (−12.55, −0.49)	−2.04 (−3.92, −0.15)	2.07 (−2.12, 6.26)	2.53 (−0.52, 5.58)	−15.45 (−31.61, 0.72)
		*P*	0.03^*∗*^	0.03^*∗*^	0.33	0.10	0.06
		*η* ^2^	0.01	0.01	0.003	0.007	0.01
	Frequent vs. significant problem	Regression coefficient (95% CI)	−5.26 (−11.30, 0.78)	−2.11 (−4.00, −0.22)	2.04 (−2.16, 6.23)	2.64 (−0.42, 5.70)	−14.76 (−30.96, 1.44)
		*P*	0.09	0.03^*∗*^	0.34	0.09	0.07
		*η* ^2^	0.008	0.01	0.003	0.008	0.009

Controlling for gender, age, parental education, track, and having regular checkups last year. CI: confidence interval; *η*^2^: partial eta squared. ^*∗*^Statistically significant at *P* < 0.05.

## Data Availability

The SPSS data file of this study is available from the corresponding author upon request.
